# Genome-Wide RNA Sequencing Analysis in Human Dermal Fibroblasts Exposed to Low-Dose Ultraviolet A Radiation

**DOI:** 10.3390/genes13060974

**Published:** 2022-05-29

**Authors:** Jinyun Wang, Satoshi Yano, Kun Xie, Yoshihisa Ohata, Taichi Hara

**Affiliations:** Laboratory of Food and Life Science, Faculty of Human Sciences, Waseda University, Tokorozawa 359-1192, Japan; wangjinyun@ruri.waseda.jp (J.W.); s.yano3@aoni.waseda.jp (S.Y.); k.xie@kurenai.waseda.jp (K.X.); trigger.happy.ohata@gmail.com (Y.O.)

**Keywords:** ultraviolet A, low-dose UVA, biomarker, IGFBP7, Fos, differentially expressed gene, senescence-associated secretory phenotype

## Abstract

Ultraviolet A (UVA) radiation can pass through the epidermis and reach the dermal skin layer, contributing to photoaging, DNA damage, and photocarcinogenesis in dermal fibroblasts. High-dose UVA exposure induces erythema, whereas low-dose, long-term UVA exposure causes skin damage and cell senescence. Biomarkers for evaluating damage caused by low-dose UVA in fibroblasts are lacking, making it difficult to develop therapeutic agents for skin aging and aging-associated diseases. We performed RNA-sequencing to investigate gene and pathway alterations in low-dose UVA-irradiated human skin-derived NB1RGB primary fibroblasts. Differentially expressed genes were identified and subjected to Gene Ontology and reactome pathway analysis, which revealed enrichment in genes in the senescence-associated secretory phenotype, apoptosis, respiratory electron transport, and transcriptional regulation by tumor suppressor p53 pathways. Insulin-like growth factor binding protein 7 (IGFBP7) showed the lowest *p*-value in RNA-sequencing analysis and was associated with the senescence-associated secretory phenotype. Protein–protein interaction analysis revealed that Fos proto-oncogene had a high-confidence network with IGFBP7 as transcription factor of the IGFBP7 gene among SASP hit genes, which were validated using RT-qPCR. Because of their high sensitivity to low-dose UVA radiation, Fos and IGFBP7 show potential as biomarkers for evaluating the effect of low-dose UVA radiation on dermal fibroblasts.

## 1. Introduction

**Figure 1:**
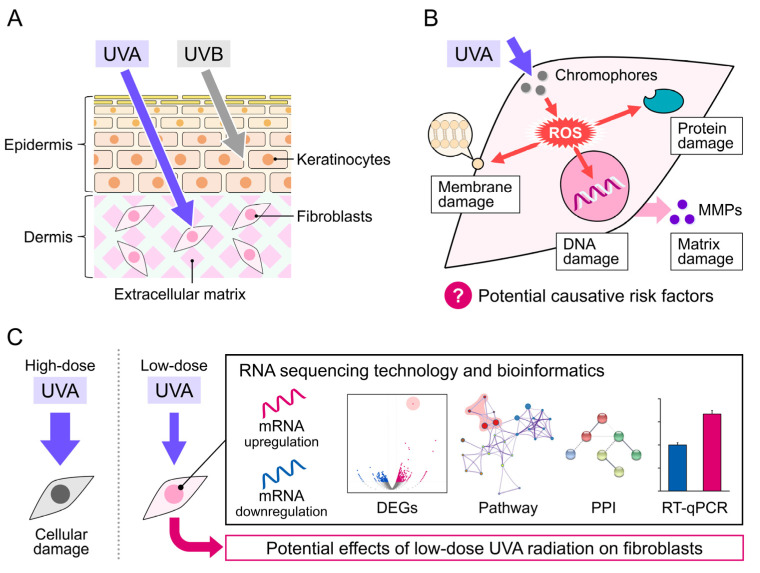
Mechanism of UVA damage to the skin. (**A**) UVA penetration into the skin dermis layers. (**B**) UVA-induced damage to cellular DNA, protein, and membrane via ROS production in skin fibroblasts. (**C**) Schematic diagram of this study.

Solar ultraviolet (UV) radiation can be divided into UVA (320–400 nm), UVB (290–320 nm), and UVC (200–290 nm). Because of their lower wavelength, most UVB and all UVC radiation is absorbed by the ozone layer, making UVA the predominant source of radiation and responsible for 95% of total residue solar UV radiation [1]. Moreover, UVA can penetrate deeper into various media, such as water, the atmosphere, or clothing, than UVB [2,3,4]. The average irradiances of UVA and UVB intensities at sea level are 5 × 10^−3^ and 0.16 × 10^−3^ J/cm^2^/s, respectively [5]. UVA crosses the epidermis to penetrate the dermis (Figure 1A) with slight but longer-lasting detrimental effects, including damage to DNA and phospholipids and cellular thymidine incorporation, leading to skin senescence and skin cell proliferation delay [5]. Additionally, UVB only reaches the epidermis, eventually causing sunburn (Figure 1A). Thus, UVA is considered as the principal contributor to photoaging and photocarcinogenesis [6]. Adaptive changes in skin cells are thought to protect the skin in response to UVA radiation; however, skin pigmentation darkening and potential detrimental effects that cannot be quickly recovered have also been observed, even after low-dose UVA exposure (≤5 J/cm^2^) [7]. Therefore, low-dose UVA-induced damage should be examined in detail. Numerous studies showed that exposure to UVA radiation at a total dosage higher than 30 J/cm^2^ for 24 h induces perceptible erythema in humans, and doses of 10 and 20 J/cm^2^ reduced HaCaT cell viability [8].Lower doses of UVA radiation (≤5 J/cm^2^) do not induce significant erythema but can induce reactive oxygen species (ROS) generation and cellular immunosuppression [9,10]. Additionally, the mechanisms of low-dose UVA radiation-caused damage are indirect, including cellular chromophore disorder and the generation of DNA-damaging ROS that affect the normal function of skin cells, making their effects difficult to evaluate and detect (Figure 1B) [11]. Thus, validated biomarkers for evaluating damage caused by low-dose UVA radiation are lacking.

The skin is composed of two parts, the epidermis and dermis, which originate from different locations in the embryo and have specific functions. Epidermal cells are often used as an in vitro model to study skin damage caused by UVA [12,13,14], because the epidermis is the first line of defense against UVA radiation. The epidermis is also the most sensitive element of the antioxidant response to UVA-induced oxidative stress, DNA damage, and photoaging [15]. In studies of UVA photoprotective products and sunscreen development, the epidermis is the most common target [16]. However, the integral gene alterations and potential effects of UVA radiation in dermal fibroblasts are unclear. A study reported that FEK4 human fibroblasts receiving over 10 J/cm^2^ of UVA radiation led to an increase in c-Fos mRNA levels [17]. Another study showed that human fibroblasts and KB cancerous cells irradiated with the same intensity of UVA presented a significant increase in expression of the AP-1 transcription factor at 1.5 h post-irradiation [18]. Additionally, UVA-radiation-induced matrix metalloproteinases (MMPs) in an in vivo mouse model, such as Swiss albino mice and SKH-1 hairless mice, which subsequently modulated epidermal keratinocytes [19,20]. These studies showed that UVA radiation induces metabolic changes in human dermal fibroblasts.

RNA sequencing (RNA-seq) technology has greatly progressed in recent decades and become a crucial transcriptome profiling tool in many aspects of research and therapy [21], including biomarker discovery and characterization of disease progression and evolution [22]. Here, we performed genome-wide RNA-seq to analyze changes in transcription factors and differentially expressed genes (DEGs) in NB1RGB normal human skin fibroblasts irradiated with low-dose UVA (at a dose rate of 5 × 10^−3^ J/cm^2^/s for 18, 36, or 72 min to achieve total doses of 5, 10, and 20 J/cm^2^, respectively) (Figure 1C). We also explored changes in the biological processes (BPs), molecular functions (MFs), and cell components (CCs) based on Gene Ontology (GO) enrichment analysis. Moreover, reactome pathway and protein–protein interaction (PPI) network analyses were performed to identify altered pathways. Candidate genes and potential damage-related biomarkers of low-dose UVA radiation were validated using RT-qPCR based on selected hub DEGs.

## 2. Materials and Methods

### 2.1. Cell Culture and UVA Irradiation

NB1RGB, human skin-derived primary fibroblasts, were purchased from the RIKEN BioResource Center (Ibaraki, Tsukuba, Japan). NB1RGB cells were cultured at 37 °C under 5% CO_2_ in minimum essential medium α containing 10% fetal bovine serum and 1% penicillin and streptomycin (168-23191, Wako Pure Chemicals Ltd., Osaka, Japan). The cells were maintained at a population doubling level of 30 to manage aging. The UVA apparatus was purchased from Analytik Jena GmbH (UVP crosslinker CL-3000L; Jena, Germany). The 365 nm wavelength bulb (8 W, 6 pieces) was set at a distance 15 cm from the bottom of the UVA apparatus. The microprocessor can modulate the radiation quantity over time to ensure sufficient UVA irradiation of the sample. UVA-treated cells seeded into a 6-well plate (Wuxi NEST Biotechnology Co., Ltd., Wuxi, China) at a density of 1 × 10^5^ cells were pre-cultured for 24 h, covered with 2 mL phosphate-buffered saline (PBS), and irradiated using the UVA apparatus at a dose rate of 5 × 10^−3^ J/cm^2^/s for 18, 36, or 72 min to achieve total doses of 5, 10, and 20 J/cm^2^, respectively. The 6-well plate was placed in the center of the apparatus during UVA irradiation. After radiation, the cells were cultured in minimum essential medium α containing 10% fetal bovine serum for another 24 h. Control cells covered with 2 mL PBS were wrapped in aluminum foil to block UVA irradiation, and then treated as in the UVA irradiation groups.

### 2.2. Cell Survival Assay

Cell survival measurement kits were purchased from Dojindo Laboratories (CCK-8; Kumamoto, Japan). Cells were seeded into 96-well plates at a density of 5 × 10^3^ cells/well, pre-cultured for 24 h at 37 °C under 5% CO_2_, washed once with PBS, and covered with 200 µL PBS per well before exposure to UVA radiation and incubation for 24 h. Cell viability was analyzed using a CCK-8 kit according to the manufacturer’s instructions. Briefly, 100 µL of medium and 10 µL of CCK-8 reagent were added to each well and incubated for 1 h at 37 °C. The absorbance of each well was measured at 450 nm using a plate reader. The cell proliferation rate was calculated from optical density measurements (corresponding to CCK-8 formazane metabolite) for UVA-exposed samples compared to in control samples (set at 100%).

### 2.3. RNA Extraction and Sequencing

Samples extracted from 6 independent wells were pooled for RNA-seq analysis. Total RNA was extracted using TRIzol™ reagent (Invitrogen, Carlsbad, CA, USA), according to the manufacturer’s protocol. The extracted RNA was quantitatively evaluated prior to cDNA library construction as follows: (1) the RNA purity was measured using a spectrophotometer (OD260/OD280), (2) 28S rRNA and 18S rRNA were analyzed using agarose electrophoresis, and (3) RNA integrity was checked using an Agilent 2100 (Agilent Technologies, Santa Clara, CA, USA). The ratio of 28S rRNA/18S rRNA, an index of RNA quality, was 2.0, and the RNA integrity number was 10, indicating high quality. mRNA was enriched using oligo (dT) beads. A cDNA library was constructed using the NEB Next^®^ Ultra™ RNA Library Prep Kit for Illumina^®^ (New England Biolabs, Ipswich, MA, USA) according to the manufacturer’s protocol. The final cDNA library was prepared after several rounds of purification, terminal repair, A-tailing, sequencing adapter ligation, size selection, and PCR enrichment. RNA sequencing was performed on the libraries by Novogene (Beijing, China) on a NovaSeq 6000 System. Reads were mapped to reference sequences using TopHat 2.

### 2.4. Bioinformatic Analysis

METASCAPE (https://metascape.org/gp/index.html#/main/step1, accessed on 5 December 2021) was used for systematic analysis. All raw data were submitted and annotated using existing human gene databases. Predicted target genes were analyzed using GO enrichment analysis of BPs, CCs, and MFs with the following criteria: *p* < 0.01, minimum count = 3, and enrichment factor > 1.5. Genes coding for protein and process pathways were evaluated in reactome pathway analysis.

### 2.5. Reverse Transcription and RT-qPCR

RNA was extracted using a ReliaPrepTM RNA cell Miniprep system (Promega, Madison, WI, USA). The extracted total RNA was reverse-transcribed into cDNA using the ReverTra Ace^®^ qPCR RT Master Mix with a gDNA remover kit (TOYOBO, Osaka, Japan). Three biological replicates were examined to ensure reproducibility. The RNA concentrations used for cDNA synthesis were 527.0, 629.4, and 96.3 ng/μL in the control groups and 524.8, 750.7, and 92.5 ng/μL in the UVA irradiation groups, respectively. IGFBP7 and Fos expression was measured using RT-qPCR with the following reaction mixture: 12.5 μL of TB Green^®^ Premix Ex Taq II (Takara, Shiga, Japan), 2 μL of cDNA, 1 μL of 10 μM 1:1 forward and reverse target primers, and 8.5 μL of nuclease-free water. Using a Thermal Cycler Dice^®^ Real Time System III (Takara, Shiga, Japan), the thermal cycling conditions were an initial denaturation step at 95 °C for 30 s, followed by 40 cycles at 95 °C for 5 s and 60 °C for 30 s. The primers for IGFBP7, Fos, and GAPDH are listed in Table 1. A single peak was observed for all amplicons in melt curve analysis. Gene expression was normalized to the geometric mean of GAPDH as an internal control, which was used as a representative housekeeping gene along with ACTB and 18S ribosomal RNA [23].

### 2.6. Statistical Analysis

The results are expressed as the mean ± standard error of the mean (SEM). Significant differences between treatments were analyzed using two-tailed Student’s *t*-tests. Significant differences (*p* < 0.05) are indicated using asterisks.

## 3. Results

### 3.1. Morphology and Proliferation Rate of NB1RGB Fibroblasts Irradiated with UVA

**Figure 2:**
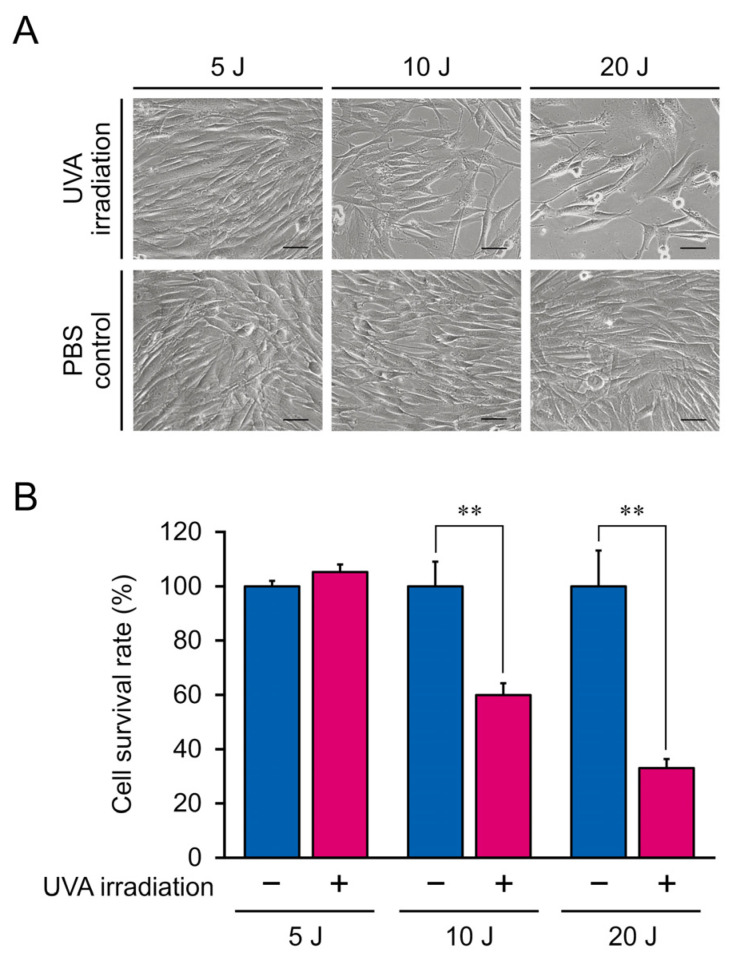
(**A**) Morphological characteristics of NB1RGB fibroblasts irradiated with 5, 10, or 20 J/cm^2^ ultraviolet A (UVA). Inset scale bar, 50 μm. (**B**) Optical density of NB1RGB fibroblasts irradiated with 5, 10, or 20 J/cm^2^ UVA. The mean ± SEM of a representative result from independent experiments performed in quadruplicate. Asterisks indicate significant differences analyzed with Student’s *t*-test, ** *p* < 0.01 vs. UVA non-irradiation group as a control at different UVA doses.

Excessive UVA radiation induces notable changes in cellular morphology and causes cell death [24,25]. Therefore, we measured the proliferation rate of NB1RGB fibroblasts irradiated doses of 5, 10, and 20 J/cm^2^, respectively. The cell proliferation rate was measured using the CCK-8 colorimetric assay. Data for irradiated samples are expressed as normalized values (corresponding control values set at 100%) and represent the SEM. Although cells treated with 5 J/cm^2^ UVA showed no morphological changes, those irradiated with 10 and 20 J/cm^2^ UVA exhibited a longer spindle shape (Figure 2A). Similarly, cell proliferation did not significantly differ between control cells and those irradiated with 5 J/cm^2^ UVA (*p* > 0.05; Figure 2B) but was significantly lower in cells irradiated with 10 and 20 J/cm^2^ UVA (*p* < 0.05).

### 3.2. RNA-seq Analysis of DEGs

**Figure 3:**
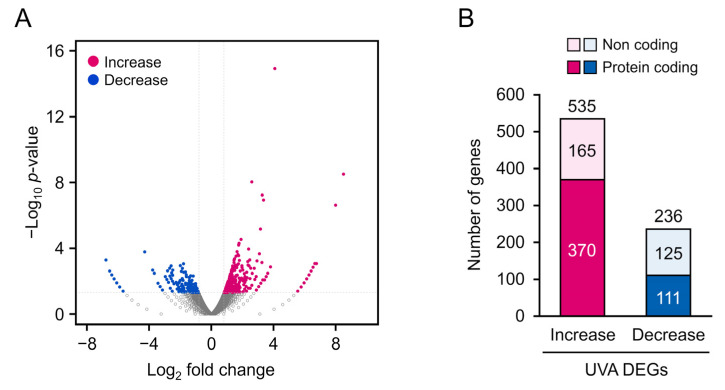
(**A**) Volcano map of 771 differentially expressed genes (DEGs) (*p* < 0.05, log_2_ fold-change > 0.8). Significantly upregulated and downregulated DEGs are represented as blue and red dots, respectively. (**B**) Distribution of DEGs.

To detect alterations in gene expression induced by 5 J/cm^2^ UVA radiation, we extracted total RNA from cells following the 5 J/cm^2^ and control treatments, and then performed RNA-seq analysis and gene annotation in comparison with published human gene databases (Metascape). In total, 12,239 clear reads were successfully filtered from the 24,172 sequenced genes and mapped onto the human genome. The annotated genes are displayed in a volcano plot (Figure 3A) to identify upregulated (blue) and downregulated (red) genes. We identified 771 DEGs (*p* < 0.05, |log_2_ fold-change| > 0.8; Figure 3B), of which 535 and 236 genes were up- and downregulated, respectively. DEGs for long or short noncoding RNAs were excluded, and the remaining 370 upregulated and 111 downregulated protein-coding DEGs were further analyzed.

### 3.3. GO Enrichment and Reactome Pathway Analysis

**Figure 4:**
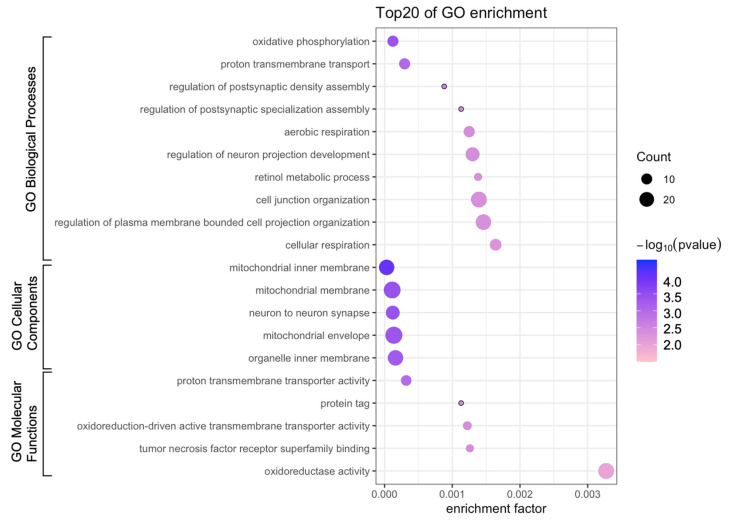
Bubble diagram of Gene Ontology (GO) enrichment analysis. Bubble size represents the number of differentially expressed genes.

**Figure 5:**
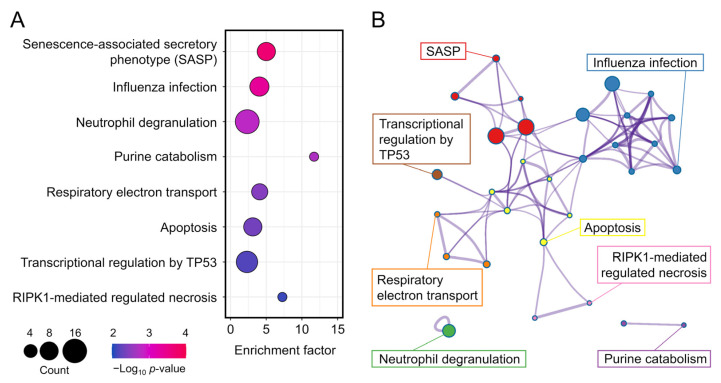
(**A**) Bubble diagram of top eight enriched reactomes. Bubble size indicates the number of differentially expressed genes. Bubble color indicates the *p*-value. (**B**) Cluster diagram of enriched pathways.

To investigate differences in the BPs, MFs, and CCs between the 5 J/cm^2^ UVA-treated and control cells, we conducted GO enrichment analysis. The most enriched BPs were oxidative phosphorylation, proton transmembrane, and regulation of neuron projection development (Figure 4, Table 2), whereas the most enriched CCs were the mitochondrial membrane, inner mitochondrial membrane, and mitochondrial envelope. The most enriched MFs were proton transmembrane transporter activity and oxidoreductase activity. We also performed reactome pathway analysis to identify pathways most strongly correlated with the significant BPs. As shown in Figure 5 and Table 3, the upregulated DEGs were significantly enriched in the senescence-associated secretory phenotype (SASP), apoptosis, respiratory electron transport, and transcriptional regulation by TP53. Because the DEGs associated with the SASP were the most abundant, they were selected for further analysis.

### 3.4. PPI Analysis of SASP-Related Genes

**Figure 6:**
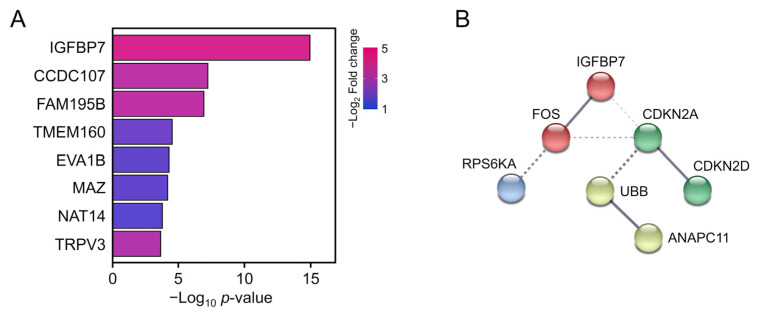
(**A**) Top eight annotated differentially expressed genes. Bar color represents the log_2_ fold-change value. Bar length represents the *p*-value. (**B**) Schematic of protein–protein interactions. Solid lines represent proven relationships. Dotted lines indicate putative associations.

To identify hub DEGs involved in the SASP, we performed PPI network analysis. *CDKN2A*, *CDKN2D*, *Fos*, *IGFBP7*, *RPS6KA1*, *UBB*, *H2BC4*, and *ANAPC11* were the most significant DEGs associated with the SASP (Table 4), among which *IGFBP7* showed the lowest *p*-value at DEGs of protein coding genes (Figure 6A). Although UBB and H2BC4 also had significant *p* and log fold-change values, they play roles in ubiquitination and were therefore shown as activated in most pathways in GO analysis, which are not suitable as biomarkers. Furthermore, protein–protein interaction analysis revealed that Fos proto-oncogene (Fos) had a high-confidence network with IGFBP7 among SASP hit genes (Figure 6B). Fos is a transcriptional factor that directly activates IGFBP7 and is used as a candidate marker gene [26]. Based on our RNA-seq data, IGFBP7 and Fos show potential for evaluating the progression of UVA irradiation-mediated damage.

### 3.5. Biomarker Validation

**Figure 7:**
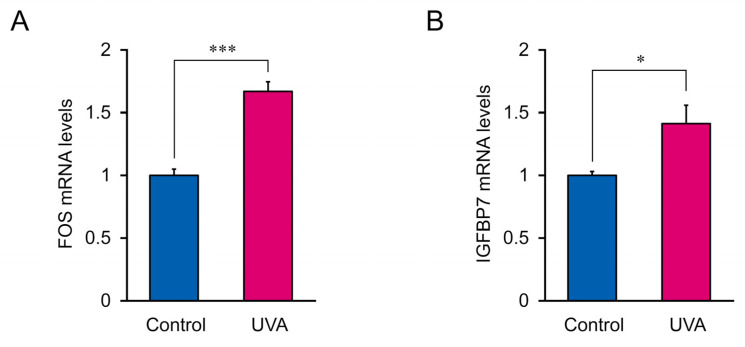
*IGFBP7* and *Fos* mRNA expression in cells treated with 5 J/cm^2^ UVA radiation. (**A**) *Fos* mRNA levels. (**B**) *IGFBP7* mRNA levels. Data represent the mean ± SEM of three biological independent experiments performed in triplicate. Asterisks indicate significant difference analyzed using Student’s *t*-test, * *p* < 0.05, *** *p* < 0.001 vs. control, UVA non-irradiation group.

To validate the putative biomarkers filtered from RNA-seq, *Fos*, and *IGFBP7*, which were the most significantly altered genes in response to low-dose UVA radiation, were analyzed using RT-qPCR. *IGFBP7* and *Fos* mRNA expression was significantly upregulated following low-dose UVA exposure, supporting the RNA-seq results (Figure 7). Thus, Fos and IGFBP7 are candidate biomarkers for monitoring damage caused by low-dose UVA radiation.

## 4. Discussion

UVA-radiation-induced cellular damage is highly correlated with the radiation intensity and dose. A lower dose but higher total intensity of UVA radiation causes more severe lipid peroxidation and oxidation-related gene alteration compared to the effects of high-dose UVA exposure, indicating that accumulated low-dose UVA results in a higher level of ROS generation and thus indirectly causes DNA damage [8,27]. Moreover, a clinical trial demonstrated that UVA radiation doses of over 30 J/cm^2^ maintained for 24 h can induce perceptible erythema in humans and that lower doses did not induce significant erythema but caused potential skin damage [9]. In HaCaT cells, irradiation with 10 and 20 J/cm^2^ UVA significantly reduced cell viability [8], whereas UVA ≤ 5 J/cm^2^ caused persistent genomic instability [28]. Consistently, in our study, 10 and 20 J/cm^2^ UVA radiation significantly reduced the viability of NB1RGB fibroblasts, indicating that dermal fibroblasts and keratinocytes have a similar tolerance to UVA radiation [8]. Furthermore, 5 J/cm^2^ UVA radiation did not significantly cause cell death and alter the cellular morphology, but the mRNA expression of 771 genes was significantly altered in NB1RGB fibroblasts.

The DEGs were enriched in the process of oxidative phosphorylation and mitochondrial inner membrane, suggesting that UVA induces mitochondrial dysfunction; most endogenous ROS are derived from the mitochondrial electron transport chain as a byproduct of oxidative phosphorylation [29]. Thus, UVA-induced ROS overproduction may result from impairments in the mitochondrial electron transport chain and cellular photodamage [30]. Moreover, mitochondrial dysfunction and the associated ROS generation contribute to altering the intracellular redox balance and are accompanied by mitochondrial translocation of p53, subsequently leading to the excretion of SASP and finally resulting in oncogene-induced cellular senescence [31,32,33].

UVA radiation induces aging of the human epidermis, even at low doses of 1–4 J/cm^2^, by influencing PI3K/AKT mediated arresting in S phase of the cell cycle [34]. However, the contribution of specific cell types to this process, particularly the role of human dermal fibroblasts, remains poorly understood. A previous study showed that UVA radiation induced oxidative stress in mouse dermal cells, and that dermal cells secreted matrix metalloproteinase-1 to modulate epidermal keratinocytes [19], suggesting that senescent cells can induce paracrine senescence in normal neighboring cells via secretion of SASP factors [35]. Moreover, chronic exposure to SASP has been shown to impair the regenerative capacity of keratinocytes in mice [36]. In our study, low-dose UVA also induced a significant alteration in SASP-related genes in NB1RGB fibroblasts, suggesting that low-dose UVA radiation accelerates human dermal fibroblast senescence, and activated SASP may interfere with the regenerative capacity of epidermal cells. Additionally, several studies showed that NF-κB is involved in regulating the SASP, as inflammatory responses can recruit macrophages to eliminate potentially senescent cells [37,38]. Thus, low-dose UVA exposure may lead to photoaging of both dermal and epidermal cells and even result in skin inflammation.

Cell senescence caused by UVA radiation is the hallmark of UVA-induced skin damage. SASP is a form of growth arrest in which senescent cells produce extracellular secretomes that mediate the senescence response of neighboring cells [39]. Inflammatory cytokines (e.g., IL-6, IL-7, IL-15), growth modulators (e.g., FGF7), angiogenic factors (e.g., angiogenin), and matrix metalloproteinases (e.g., MMP-1, MMP-3, MMP-10) are all associated with the SASP [40]. Studies showed that cellular responses and stress sensors that regulate the SASP are influenced by UVA radiation [41,42]. UVA radiation induces the generation of ROS, including superoxide (O_2_•- or singlet oxygen (^1^O_2_)), which can oxidize cellular proteins, making them relatively insoluble [43]. Intracellular deposition of insoluble oxidized protein aggregates is associated with SASP induction and inflammatory disorders [44,45]. UVA-induced cell senescence may be strongly associated with SASP activation. Because various factors also induce SASP responses to clear antigens, toxins, and cancer cells, not all SASP-related genes reflect the damage caused by low-dose UVA radiation [46]. Of the SASP-related DEGs identified in this study, *IGFBP7* showed the most significant *p*-value. IGFBP family members are not only highly involved in the SASP, but IGFBP4/7 are also key components required to trigger cell senescence [47]. Similarly, a genome-wide shRNA screening of melanocytes showed that IGFBP7 plays a central role in senescence and apoptosis [26]. Together, these findings indicate that cells sensitively express IGFBP7 during UVA-radiation-induced senescence, and IGFBP7 is a potential biomarker for monitoring low-dose UVA-induced cell damage. Additionally, we found that UVA promotes activation of the oxidative stress-sensitive signaling protein p38 and its downstream target Fos. The *IGFBP7* promoter contains a consensus binding site for the dimeric AP-1 transcription factor, including c-Fos and c-Jun [26]. Fos mRNA was significantly regulated in senescent fibroblasts. Similar results were reported for the HaCaT cell line [48,49,50], suggesting that the mRNA expression of *Fos* can indicate both senescence and inflammation induced by UVA [51,52,53]. Thus, Fos is another potential biomarker of low-dose UVA-induced cell damage in both epidermal and dermal cells.

## 5. Conclusions

This study characterized genetic changes and pathway alterations induced by low-dose UVA radiation in NB1RGB fibroblasts using RNA-seq; low-dose UVA-induced DEGs were mostly enriched in mitochondrial dysfunction and SASP activation. Fos and IGFBP7, which respond to inflammation and cell senescence, show potential as biomarkers of damage caused by low-dose UVA radiation.

## Figures and Tables

**Table 1:** Primer sequences for real-time qPCR.

Genes	Primer Sequence (5′-3′)	Size (bp)
GAPDH	Forward 5′-GAAGGTGAAGGTCGGAGTCA-3′	290
	Reverse 5′-TGGACTCCACGACGTACTCA-3′	
IGFBP7	Forward 5′-TTGAGCTGTGAGGTCATCGG-3′	188
	Reverse 5′-TCCTTACTTAGAGGAGATACCAGCA-3′	
Fos	Forward 5′-TGTGAAGACCATGACAGGAGG-3′	181
	Reverse 5′-TTGGTCTGTCTCCGCTTGG-3′	

**Table 2:** Gene Ontology (GO) analysis of low-dose UVA-related differentially expressed genes.

	Go Number	Description	Log *p* Value
Biological processes	GO: 0006119	oxidative phosphorylation	−3.91
	GO: 1902600	proton transmembrane transport	−3.53
	GO: 0099151	regulation of postsynaptic density assembly	−3.06
	GO: 0099150	regulation of postsynaptic specialization assembly	−2.95
	GO: 0009060	aerobic respiration	−2.90
	GO: 0010975	regulation of neuron projection development	−2.89
	GO: 0042572	retinol metabolic process	−2.86
	GO: 0034330	cell junction organization	−2.86
Cellular components	GO: 0005743	mitochondrial inner membrane	−4.51
	GO: 0031966	mitochondrial membrane	−3.95
	GO: 0098984	neuron to neuron synapse	−3.92
	GO: 0005740	mitochondrial envelope	−3.86
	GO: 0019866	organelle inner membrane	−3.79
Molecular functions	GO: 0015078	proton transmembrane transporter activity	−3.50
	GO: 0031386	protein tag	−2.95
	GO: 0015453	oxidoreduction-driven active transmembrane transporter activity	−2.91
	GO: 0032813	tumor necrosis factor receptor superfamily binding	−2.90
	GO: 0016491	oxidoreductase activity	−2.49

**Table 3:** Reactome analysis of low-dose UVA-related differentially expressed genes.

Reactome ID	Description	Log *p*-Value	Enrichment	Gene Symbol
R-HSA-2559582	Senescence-associated secretory phenotype	−3.69	5.02	*CDKN2A*, *CDKN2D*, *Fos*, *IGFBP7*, *RPS6KA1*, *UBB*, *H2BC4*, *ANAPC11*
R-HSA-168255	Influenza infection	−3.38	4.05	*POLR2F*, *POLR2L*, *RPL8*, *RPL36AL*, *RPL41*, *RPS25*, *PABPN1*, *ISG15*, *RPL36*
R-HSA-6798695	Neutrophil degranulation	−2.80	2.34	*APRT*, *ATP6V0C*, *CYBA*, *CFD*, *FTH1*, *PKP1*, *RAB3A*, *SLC2A5*, *KCNAB2*, *DPP7*, *RAB4B*, *RAB24*, *CD177*, *TXNDC5*, *GHDC*, *NAPRT*
R-HSA-74259	Purine catabolism	−2.70	11.70	*NUDT1*, *DNPH1*, *NT5C*
R-HSA-611105	Respiratory electron transport	−2.44	4.09	*COX6B1*, *COX8A*, *NDUFA1*, *NDUFA3*, *UQCRH*, *COX14*
R-HSA-109581	Apoptosis	−2.36	3.12	*CDKN2A*, *PKP1*, *PSMB10*, *UBB*, *SEM1*, *TRADD*, *FADD*, *BBC3*
R-HSA-370098	Transcriptional regulation by TP53	−2.19	2.31	*CDKN2A*, *COX6B1*, *COX8A*, *Fos*, *PIN1*, *POLR2F*, *POLR2L*, *STK11*, *UBB*, *BBC3*, *PRELID1*, *COX14*
R-HSA-5213460	RIPK1-mediated regulated necrosis	−2.10	7.27	*UBB*, *TRADD*, *FADD*

**Table 4:** Differentially expressed genes in the senescence-associated secretory phenotype pathway.

Gene	Description	Log2 Fold-Change	*p*-Value
*IGFBP7*	Regulation of cell growth, signal transduction	4.1	1.2 × 10^−15^
*UBB*	Modification-dependent protein catabolic process	1.3	0.0022
*H2BC4*	Core component of nucleosome	1.8	0.012
*Fos*	RNA polymerase II binding	1	0.024
*CDKN2A*	Cyclin-dependent kinase inhibitor	0.95	0.026
*RPS6KA1*	Ribosomal protein S6 kinase	1.2	0.028
*ANAPC11*	Ubiquitin-dependent protein catabolic process	0.89	0.034
*CDKN2D*	Cyclin-dependent kinase 4 inhibitor	0.86	0.05

## Data Availability

The data presented in this study are available on request from the corresponding author.

## References

[1] Sliney D.H. (2007). Radiometric quantities and units used in photobiology and photochemistry: Recommendations of the Commission Internationale de L’Eclairage (International Commission on Illumination). Photochem. Photobiol..

[2] Schmalwieser A.W., Klotz B., Schwarzmann M., Baumgartner D.J., Schreder J., Schauberger G., Blumthaler M. (2019). The Austrian UVA-Network. Photochem. Photobiol..

[3] D’Orazio J., Jarrett S., Amaro-Ortiz A., Scott T. (2013). UV radiation and the skin. Int. J. Mol. Sci..

[4] Stunova A., Vistejnova L. (2018). Dermal fibroblasts-A heterogeneous population with regulatory function in wound healing. Cytokine Growth Factor Rev..

[5] D’Angelo S., Ingrosso D., Perfetto B., Baroni A., Zappia M., Lobianco L.L., Tufano M.A., Galletti P. (2001). UVA irradiation induces L-isoaspartyl formation in melanoma cell proteins. Free Radic. Biol. Med..

[6] Battie C., Jitsukawa S., Bernerd F., Del Bino S., Marionnet C., Verschoore M. (2014). New insights in photoaging, UVA induced damage and skin types. Exp. Derm..

[7] Dissanayake N.S., Greenoak G.E., Mason R.S. (1993). Effects of ultraviolet irradiation on human skin-derived epidermal cells in vitro. J. Cell. Physiol..

[8] Shorrocks J., Paul N.D., McMillan T.J. (2008). The Dose Rate of UVA Treatment Influences the Cellular Response of HaCaT Keratinocytes. J. Investig. Derm..

[9] Welti M., Ramelyte E., Dummer R., Imhof L. (2020). Evaluation of the minimal erythema dose for UVB and UVA in context of skin phototype and nature of photodermatosis. Photodermatol. Photoimmunol. Photomed..

[10] Halliday G.M., Rana S. (2008). Waveband and dose dependency of sunlight-induced immunomodulation and cellular changes. Photochem. Photobiol..

[11] Brem R., Guven M., Karran P. (2017). Oxidatively-generated damage to DNA and proteins mediated by photosensitized UVA. Free Radic. Biol. Med..

[12] Traynor N.J., Beattie P.E., Ibbotson S.H., Moseley H., Ferguson J., Woods J.A. (2005). Photogenotoxicity of hypericin in HaCaT keratinocytes: Implications for St. John’s Wort supplements and high dose UVA-1 therapy. Toxicol. Lett..

[13] Robinson K.S., Traynor N.J., Moseley H., Ferguson J., Woods J.A. (2010). Cyclobutane pyrimidine dimers are photosensitised by carprofen plus UVA in human HaCaT cells. Toxicol. Vitr..

[14] Yin J.J., Liu J., Ehrenshaft M., Roberts J.E., Fu P.P., Mason R.P., Zhao B. (2012). Phototoxicity of nano titanium dioxides in HaCaT keratinocytes--generation of reactive oxygen species and cell damage. Toxicol. Appl. Pharm..

[15] Ou-Yang H., Stamatas G., Saliou C., Kollias N. (2004). A chemiluminescence study of UVA-induced oxidative stress in human skin in vivo. J. Investig. Derm..

[16] Meloni M., Farina A., de Servi B. (2010). Molecular modifications of dermal and epidermal biomarkers following UVA exposures on reconstructed full-thickness human skin. Photochem. Photobiol. Sci..

[17] Bose B., Soriani M., Tyrrell R.M. (1999). Activation of expression of the c-fos oncogene by UVA irradiation in cultured human skin fibroblasts. Photochem. Photobiol..

[18] Soriani M., Hejmadi V., Tyrrell R.M. (2000). Modulation of c-jun and c-fos transcription by UVB and UVA radiations in human dermal fibroblasts and KB cells. Photochem. Photobiol..

[19] Lan C.-C.E., Hung Y.-T., Fang A.-H., Ching-Shuang W. (2019). Effects of irradiance on UVA-induced skin aging. J. Derm. Sci..

[20] Kandan P.V., Balupillai A., Kanimozhi G., Khan H.A., Alhomida A.S., Prasad N.R. (2020). Opuntiol Prevents Photoaging of Mouse Skin Blocking Inflammatory Responses and Collagen Degradation. Oxidative Med. Cell. Longev..

[21] Hong M., Tao S., Zhang L., Diao L.T., Huang X., Huang S., Xie S.J., Xiao Z.D., Zhang H. (2020). RNA sequencing: New technologies and applications in cancer research. J. Hematol. Oncol..

[22] Stark R., Grzelak M., Hadfield J. (2019). RNA sequencing: The teenage years. Nat. Rev. Genet..

[23] Livak K.J., Schmittgen T.D. (2001). Analysis of relative gene expression data using real-time quantitative PCR and the 2(-Delta Delta C(T)) Method. Methods.

[24] Gilchrest B.A. (2013). Photoaging. J. Investig. Derm..

[25] Kammeyer A., Luiten R.M. (2015). Oxidation events and skin aging. Ageing Resh. Rev..

[26] Wajapeyee N., Serra R.W., Zhu X., Mahalingam M., Green M.R. (2008). Oncogenic BRAF induces senescence and apoptosis through pathways mediated by the secreted protein IGFBP7. Cell.

[27] Kerscher M., Volkenandt M., Gruss C., Reuther T., von Kobyletzki G., Freitag M., Dirschka T., Altmeyer P. (1998). Low-dose UVA phototherapy for treatment of localized scleroderma. J. Am. Acad. Derm..

[28] Phillipson R.P., Tobi S.E., Morris J.A., McMillan T.J. (2002). UV-A induces persistent genomic instability in human keratinocytes through an oxidative stress mechanism. Free Radic. Biol. Med..

[29] Pillai S., Oresajo C., Hayward J. (2005). Ultraviolet radiation and skin aging: Roles of reactive oxygen species, inflammation and protease activation, and strategies for prevention of inflammation-induced matrix degradation–a review. Int. J. Cosmet. Sci..

[30] Guidot D.M., McCord J.M., Wright R.M., Repine J.E. (1993). Absence of electron transport (Rho 0 state) restores growth of a manganese-superoxide dismutase-deficient Saccharomyces cerevisiae in hyperoxia. Evidence for electron transport as a major source of superoxide generation in vivo. J. Biol. Chem..

[31] Johmura Y., Nakanishi M. (2016). Multiple facets of p53 in senescence induction and maintenance. Cancer Sci..

[32] Beauséjour C.M., Krtolica A., Galimi F., Narita M., Lowe S.W., Yaswen P., Campisi J. (2003). Reversal of human cellular senescence: Roles of the p53 and p16 pathways. EMBO J..

[33] Narita M., Narita M., Krizhanovsky V., Nuñez S., Chicas A., Hearn S.A., Myers M.P., Lowe S.W. (2006). A novel role for high-mobility group a proteins in cellular senescence and heterochromatin formation. Cell.

[34] He Y.-Y., Council S.E., Feng L., Chignell C.F. (2008). UVA-induced cell cycle progression is mediated by a disintegrin and metalloprotease/epidermal growth factor receptor/AKT/Cyclin D1 pathways in keratinocytes. Cancer Res..

[35] Acosta J.C., Banito A., Wuestefeld T., Georgilis A., Janich P., Morton J.P., Athineos D., Kang T.W., Lasitschka F., Andrulis M. (2013). A complex secretory program orchestrated by the inflammasome controls paracrine senescence. Nat. Cell Biol..

[36] Ritschka B., Storer M., Mas A., Heinzmann F., Ortells M.C., Morton J.P., Sansom O.J., Zender L., Keyes W.M. (2017). The senescence-associated secretory phenotype induces cellular plasticity and tissue regeneration. Genes Dev..

[37] Kuilman T., Michaloglou C., Vredeveld L.C., Douma S., van Doorn R., Desmet C.J., Aarden L.A., Mooi W.J., Peeper D.S. (2008). Oncogene-induced senescence relayed by an interleukin-dependent inflammatory network. Cell.

[38] Orjalo A.V., Bhaumik D., Gengler B.K., Scott G.K., Campisi J. (2009). Cell surface-bound IL-1alpha is an upstream regulator of the senescence-associated IL-6/IL-8 cytokine network. Proc. Natl. Acad. Sci. USA.

[39] Gorgoulis V., Adams P.D., Alimonti A., Bennett D.C., Bischof O., Bishop C., Campisi J., Collado M., Evangelou K., Ferbeyre G. (2019). Cellular Senescence: Defining a Path Forward. Cell.

[40] Coppé J.-P., Desprez P.-Y., Krtolica A., Campisi J. (2010). The senescence-associated secretory phenotype: The dark side of tumor suppression. Annu. Rev. Pathol..

[41] Birch J., Gil J. (2020). Senescence and the SASP: Many therapeutic avenues. Genes Dev..

[42] Sha J., Arbesman J., Harter M.L. (2020). Premature senescence in human melanocytes after exposure to solar UVR: An exosome and UV-miRNA connection. Pigment. Cell Melanoma. Res..

[43] Cadet J., Douki T., Ravanat J.L. (2015). Oxidatively generated damage to cellular DNA by UVB and UVA radiation. Photochem. Photobiol..

[44] Davies M.J. (2003). Singlet oxygen-mediated damage to proteins and its consequences. Biochem. Biophys. Res. Commun..

[45] Krisko A., Radman M. (2013). Phenotypic and genetic consequences of protein damage. PLoS Genet..

[46] Ren J.L., Pan J.S., Lu Y.P., Sun P., Han J. (2009). Inflammatory signaling and cellular senescence. Cell Signal..

[47] Severino V., Alessio N., Farina A., Sandomenico A., Cipollaro M., Peluso G., Galderisi U., Chambery A. (2013). Insulin-like growth factor binding proteins 4 and 7 released by senescent cells promote premature senescence in mesenchymal stem cells. Cell Death Dis..

[48] Silvers A.L., Bachelor M.A., Bowden G.T. (2003). The role of JNK and p38 MAPK activities in UVA-induced signaling pathways leading to AP-1 activation and c-Fos expression. Neoplasia.

[49] Salama S.A., Arab H.H., Omar H.A., Gad H.S., Abd-Allah G.M., Maghrabi I.A., Al Robaian M.M. (2018). L-carnitine mitigates UVA-induced skin tissue injury in rats through downregulation of oxidative stress, p38/c-Fos signaling, and the proinflammatory cytokines. Chem. Biol. Interact..

[50] Oh J.H., Joo Y.H., Karadeniz F., Ko J., Kong C.S. (2020). Syringaresinol Inhibits UVA-Induced MMP-1 Expression by Suppression of MAPK/AP-1 Signaling in HaCaT Keratinocytes and Human Dermal Fibroblasts. Int. J. Mol. Sci..

[51] Stein B., Baldwin A.S., Ballard D.W., Greene W.C., Angel P., Herrlich P. (1993). Cross-coupling of the NF-kappa B p65 and Fos/Jun transcription factors produces potentiated biological function. EMBO J..

[52] Min W., Bin Z.W., Quan Z.B., Hui Z.J., Sheng F.G. (2009). The signal transduction pathway of PKC/NF-kappa B/c-fos may be involved in the influence of high glucose on the cardiomyocytes of neonatal rats. Cardiovasc. Diabetol..

[53] Irving J., Feng J., Wistrom C., Pikaart M., Villeponteau B. (1992). An altered repertoire of fos/jun (AP-1) at the onset of replicative senescence. Exp. Cell Res..

